# Taxonomic study of *Thiotricha* Meyrick (Lepidoptera, Gelechiidae) in Japan, with the description of two new species

**DOI:** 10.3897/zookeys.897.38529

**Published:** 2019-12-09

**Authors:** Khine Mon Mon Kyaw, Sadahisa Yagi, Jouhei Oku, Yositaka Sakamaki, Toshiya Hirowatari

**Affiliations:** 1 Entomological Laboratory, Graduate School of Bioresource and Bioenvironmental Sciences, Kyushu University, 744 Motooka, Nishi-ku, Fukuoka, 819-0395, Japan; 2 Entomological Laboratory, Graduate School of Agriculture, Kagoshima University, 1-21-24 Korimoto, Kagoshima, 890-0065, Japan; 3 Entomological Laboratory, Faculty of Agriculture, Kyushu University, 744 Motooka, Nishi-ku, Fukuoka, 819-0395, Japan

**Keywords:** distribution, host plants, morphology, new species, portable case, pupa, taxonomy

## Abstract

A part of Japanese species of the genus *Thiotricha* Meyrick, 1886 are reviewed. Three species described by Omelko (1984) in the genus *Cnaphostola* Meyrick, 1918 are placed in combination with *Thiotricha*; *Thiotricha biformis*, *T. angustella***comb. nov.** and *T. venustalis***comb. nov.** These species are redescribed, and two new species, *T. elaeocarpiella* Kyaw, Yagi & Hirowatari, **sp. nov.** and *T. flavitermina* Kyaw, Yagi & Hirowatari, **sp. nov.** are described based on external morphological characters of adults and genitalia of males and females. *Thiotricha chujaensis* (Park, 2016), **comb. nov.** described in *Cnaphostola* from Korea, is newly recorded in Japan feeding on *Mallotus japonica* (Euphorbiaceae). One of the new species, *T. elaeocarpiella***sp. nov.** has been associated with two different plants, *Elaeocarpus zollingeri* (Elaeocarpaceae) and *Rhaphiolepis indica* (Rosaceae). This paper presents the ﬁrst comprehensive description of the morphology, pupal morphology and biology of species previously treated in the genus *Cnaphostola* and their relatives in Japan.

## Introduction

The family Gelechiidae is one of the largest families of Microlepidoptera in the world and includes more than 4,700 described species belonging to approximately 500 genera (van Nieukerken et al. 2011). In the Palearctic region, there are more than 1,500 species (Piskunov 1990). In Japan, 288 species of this family have been recorded, including 41 unidentified species (Jinbo 2016). There is a great number of species that remain to be described, as Gelechiidae is one of the least studied Lepidoptera families. Currently, this family is thought to consist of seven subfamilies: Anacampsinae, Dichomeridinae, Apatetrinae, Thiotrichinae, Anomologinae, Gelechiinae, and Physoptilinae, mainly based on molecular analyses (Karsholt et al. 2013).

The subfamily Thiotrichinae includes the genera *Thiotricha* Meyrick, 1886, *Macrenches* Meyrick, 1904, *Palumbina* Rondani, 1876 and *Polyhymno* Chambers, 1874. Among them, *Thiotricha* Meyrick, 1886 and *Polyhymno* Chambers, 1874 have a long taxonomic history with various opinions about their separation. The genus *Thiotricha* includes globally nearly 100 described species and is most diverse in Asia (Karsholt et al. 2013). In Japan, 15 species have been recorded and were later treated as *Polyhymno* (Suzuki and Komai 1984; Oku 2003). In 2005, Ueda and Fujiwara described the new species *Thiotricha prunifolivora* Ueda & Fujiwara, 2005 from the host plant *Symplocos prunifolia* (Family: Symplocaceae), with a biological note on the immature stages. Then, this species and the 15 previously recorded species were tentatively treated in the genus *Thiotricha*, together with a taxonomic comment that mainly follows Omelko (1999) and Park (2004). In 2009, *Thiotricha* was synonymized with *Polyhymno* by Ponomarenko (2009), without further comments. Recently, Karsholt et al. (2013) conducted a molecular analysis of the Gelechiidae without *Polyhymno* species in their analysis. However, they compared the type species, *P. longistrigella* Chambers morphologically with species of *Thiotricha*. This resulted in the recognition of both *Thiotricha* and *Polyhymno* as valid genera. Moreover, also their biology differs, the larvae of *Polyhymno* are leaf-spinners and leaf-webbers in Fabaceae (Busck 1900), but larvae of *Thiotricha*, as far as known, make a portable case and feed on flowers and seeds (Robinson et al. 1994, Ueda and Fujiwara 2005).

Another genus, *Cnaphostola* Meyrick, 1918, was described for the single species *C. adamantina* Meyrick, 1918, collected in Assam, North India. Three additional species; *C. biformis* Omelko, 1984, *C. angustella*Omelko 1984, and *C. venustalis* Omelko, 1984, were described from the Primorsky Territory in the Russian Far East (Omelko 1984). These three species have also been recorded from Japan (Oku 2003; Ueda 2013). In 2016, Park described *C. chujaensis* from Chuja Island, Korea, tentatively placing it in *Cnaphostola*. Today, therefore, this genus comprises five species in total (Park and Kim 2016). Kogi (2004, 2008) observed and reported the larval feeding of *C. venustalis* and *C. angustella* on the host plant *Quercus dentata* (Fagaceae), in Hokkaido, Japan. Otherwise, the biology of the immature stages of this genus has not been studied in detail. The members of *Cnaphostola* are very similar to those of *Thiotricha* or *Polyhymno*. Meyrick (1918) noted that the genus *Cnaphostola* probably belongs to the *Thiotricha* group; however, it was not included in the molecular analysis of Karsholt et al. (2013). Then, Sohn et al. (2015) combined *Cnaphostola biformis* as *Thiotricha biformis* without any explanation.

Although the phylogenetic relationship and synonymy of the genera *Cnaphostola*, *Thiotricha* and *Polyhymno* is not fully resolved to date, we place all Japanese species here in *Thiotricha*, we review the Japanese species and compare the morphological characters, including head parts, wing marking, venation, and genitalia, to solve the aforementioned taxonomic problems. Further, we describe two new species with photographs of male and female adults, wing venation, and genitalia. We also report the biology of immature stages of some species, the pupal morphology of *T. chujaensis* (Park, 2016) comb. nov. and *T. elaeocarpiella* Kyaw, Yagi & Hirowatari sp. nov. and discuss the larval feeding mode of this group.

## Materials and methods

Dried specimens deposited in the Entomological Laboratory, Kyushu University, Fukuoka (**ELKU**); Osaka Prefecture University, Sakai (**OPU**); the Entomological Laboratory, Kagoshima University, Kagoshima (**KGU**); the National Museum of Nature and Science, Tsukuba, Japan (**NSMT**); and T. Oku’s collection, Morioka (**TO**) were examined. Field research was also conducted across Japan from Hokkaido to Okinawa. Larval portable cases were collected from host plants and light traps were used to collect adults. External morphological characteristics were first observed under a stereo microscope (Nikon SMZ-U), and then genitalia slides were prepared as follows: the abdomen was detached, placed in a glass tube with 10% potassium hydroxide (KOH) solution, and boiled in water for ca. 10–15 mins, depending on the size of the genitalia to macerate. After that, the boiled abdomen was neutralized in acetic acid, stained with Chlorazol Black E, and then rinsed with 70% ethanol solution to remove residual scales and internal soft parts. Then, the abdomen was dissected by cutting its intersegmental membrane between T7 and T8 with fine small insect pins. The genitalia were separated and transferred to a glass plate with 50% glycerol solution for observation. After observation, the genitalia and the abdomen were dehydrated in a 70%–100% ethanol series and mounted on a microscope slide in Euparal. Photographs of adults were taken using a Leica S8APO with a digital camera (Canon EOS 7D), and focus stacking was performed in Combine ZP (Hadley, 2010). Photographs of male and female genitalia were taken using a biological microscope (Olympus BX43) with a digital camera (Olympus E5).

Unless otherwise noted, the specimens are deposited in the Entomological Laboratory of Kyushu University (**ELKU**).

## Terminology

The descriptive terminology follows Park and Kim (2016) for wing markings, and Karsholt et al. (2013) and Ueda and Fujiwara (2005) for genitalia. The scientific names of plants follow Yonekura and Kajita (2003).

## Taxonomy

### Genus Thiotricha

Taxon classificationAnimaliaLepidopteraGelechiidae

Meyrick, 1886

99EF13FE-CA8C-58B6-94AF-0F5C3F426145

#### Type species.

*Thiotricha thorybodes* Meyrick, 1886 designated by Meyrick, 1925:101.

### Thiotricha biformis

Taxon classificationAnimaliaLepidopteraGelechiidae

(Omelko, 1984)

F3B4DBC7-9438-50BC-B0DD-53F105DA7CCF

Figs 4A, B , 7A , 8A, B , 9A , 10A

Cnaphostola biformis Omelko, 1984: 32; Omelko 1999: 183; Oku 2003: 65; Park and Ponomarenko 2007: 45; Ueda 2013: 298.
Thiotricha biformis : Sohn et al. 2015: 116

#### Material examined.

Japan – **Hokkaido** [Hokkaido] • 1♂,1♀; Katsuranosawa, Uryu-cho, Uryu-gun; 20 Jul. 2018; S. Yagi leg. • 1 ♂; Katsuranosawa, Ishikari city; 15 Jul. 2007; H. Kogi leg. • 1♂; Manzi, Kurisawa; 8 Jul. 2001; H. Kogi leg.; gen. slide no. KM-143 • 1 ♂, 1♀; same locality and collector; 28 Jul. 2003; gen. slide no. KM-144(♂) • 1♂; Kotan Atuta; 10 Jul. 2003; H. Kogi leg. • 1♀; Nopporo (Atsubetsu); 27 Jul. 1993; T. Hirowatari leg. • 2♀♀; Kamisibun, Iwamizawa; 9 Jul 2011; 8 Aug. 2012; H. Kogi leg.; gen. slide no. KM-145 • 1♀; Hukui, Niseko; 4 Aug. 2006; H. Kogi leg. • 1♀; Tiyosibetu, Hamamasu; 14 Jul. 2000; H. Kogi leg. • 1♀; Asari Pass, Otaru; 28 Jul. 2012; H. Kogi leg.; gen. slide no. KM-146. – **Honshu** [Iwate] • 1♀; Atei-Sanso Niisato vill.; 6 Jul. 2002; T. Oku leg. (TO).

#### Diagnosis.

The forewing is white with broad, dark brown fascia, a rather large yellow patch before the apex, and a black rounded apical spot demarcated by a white line. The anellus lobe of the male genitalia is a short and heavily sclerotized beak-shaped lobe basally, armed with a small claw-like process apically, which is a unique characteristic of this species. The apopyhsis posterioris of the female genitalia is ca. 1/2 the length of the papilla analis and approx. two times longer than the apophysis anterioris; the signum is absent in the corpus bursae.

#### Description.

**Male.** (Figs 4A, 7A). Forewing length 3.2–3.7 mm. Wing expanse 7.0–8.1 mm.

***Head***: covered with shiny, creamy white appressed scales. Antennae filiform; basal segment (scape) elongate without pecten, creamy white; flagellum creamy white on dorsal surface before middle, then entirely grayish brown beyond on its dorsal and ventral surface, with rather long and fine cilia ventrally. Labial palpus white, long, and recurved; first segment shortest with creamy white scales; second segment thickened, ca. 2.5 the thickness times of first, covered entirely with white scales; third segment white on dorsal surface, sparse brown scales medially on ventral surface, as long as second segment, apex sharply acute.

***Thorax***: creamy white. Tegula shiny, creamy white dorsally, with brown scales along anterior margin.

***Legs***: white; forefemur, tibia, and tarsus suffused inwardly with brownish tinge, white on outer surface; mid legs entirely white; hind femur and tibia white, with a row of long, stiff, stout white bristles on upper and lower surfaces; all tarsal segments brownish gray.

***Forewing***: eleven veins, R_3_ + R_4_ stalked, M_1_ separate, R_5_ absent, anal veins furcate (Fig. 7A). Forewing broader in this genus, ground color white to middle, followed by a dark brown fascia, rather large yellow patch before apex not protruding to costal margin; small brown area in costal margin, intercepted by oblique white lines with a rounded black apical spot; small scattering of yellow scales below apical point at outer margin, small brown scale in tornal area; cilia blackish brown at inner margin of apex, outer margin with brown through inner base of wing.

***Hindwing***: darker grayish brown with tiny black apical dot at apex; cilia well-fringed, grayish brown, with dark brown at tip of wing.

***Male genitalia***: (Figs 8A, B, 9A) eighth abdominal sternite rather semicircular, broadly concave with strongly sclerotized margin. Uncus semicircular with a few hairs on top. Gnathos sickle-shaped, short and stout, wide and flattened posteriorly, and then curved at middle toward apex. Tegumen long, slightly concave medially, bearing dense hairs on dorsal surface at approx. the midpoint. Anellus lobe, a short and heavily sclerotized beak-shaped lobe, approx. half the length of valva, slightly narrow at base, and then abruptly dilate with short and small claw-like process apically, with a few sclerotized spines at approx. the midpoint on inner dorsal surface. Valva elongate, slender, and curved inwardly, somewhat broad basally, narrowly elongate from base to 2/3 of its length, then distended again at apex, bearing thin and fine setae on its inner surface. Vinculum moderately broad, median process rising to a pair of transverse ridges of short, thorn-like spines on edge of posterior surface, rather long and short fine setae emerging around and below the ridge surface. Saccus broad basally, somewhat triangularly produced. Phallus short and sclerotized, clavate basally and straightly elongate with a round tip distally.

**Figure 1.**
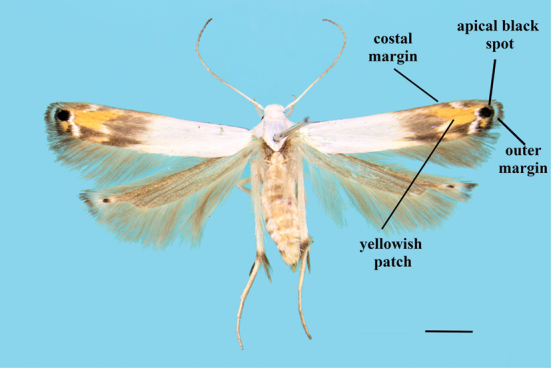
Forewing pattern elements. *Thiotricha biformis*, female. Scale bar: 1 mm.

**Female.** (Figs 1, 4B). Forewing length 2.5–3.9 mm. Wing expanse 5.5–8.2 mm. Similar to male.

***Female genitalia***: (Fig. 10A) papilla analis long and bilobed with short and long fine setae on its entire surface. Apophyses short; apophysis anterioris shorter, nearly 1/2 length of apophysis posterioris. Ostium opening near anterior margin of 8^th^ sternite. Ductus bursae partially sclerotized, narrow, and slightly elongate, as long as corpus bursae. Corpus bursae large and rounded; signum absent.

#### Distribution.

Japan (Hokkaido, Honshu), Russia, Korea.

#### Host plant.

Unknown.

#### Remarks.

Sohn et al. (2015) treated this species as *Thiotricha biformis*, without explaining the taxonomic reasons.

**Figure 2.**
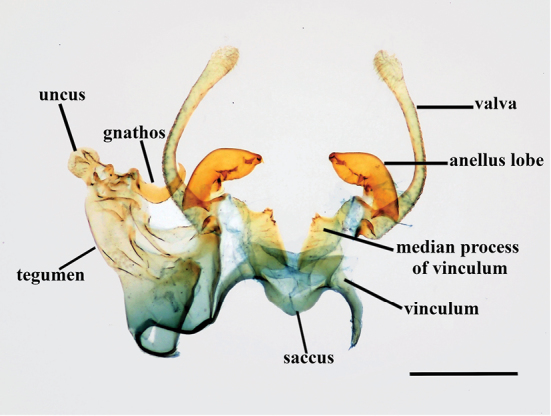
Male genitalia. *Thiotricha biformis*. Scale bar: 0.4 mm.

**Figure 3.**
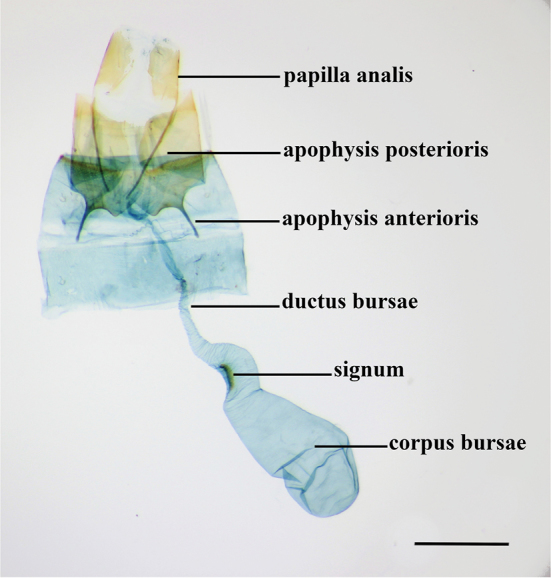
Female genitalia. *Thiotricha angustella* comb. nov. Scale bar: 0.4 mm.

**Figure 4.**
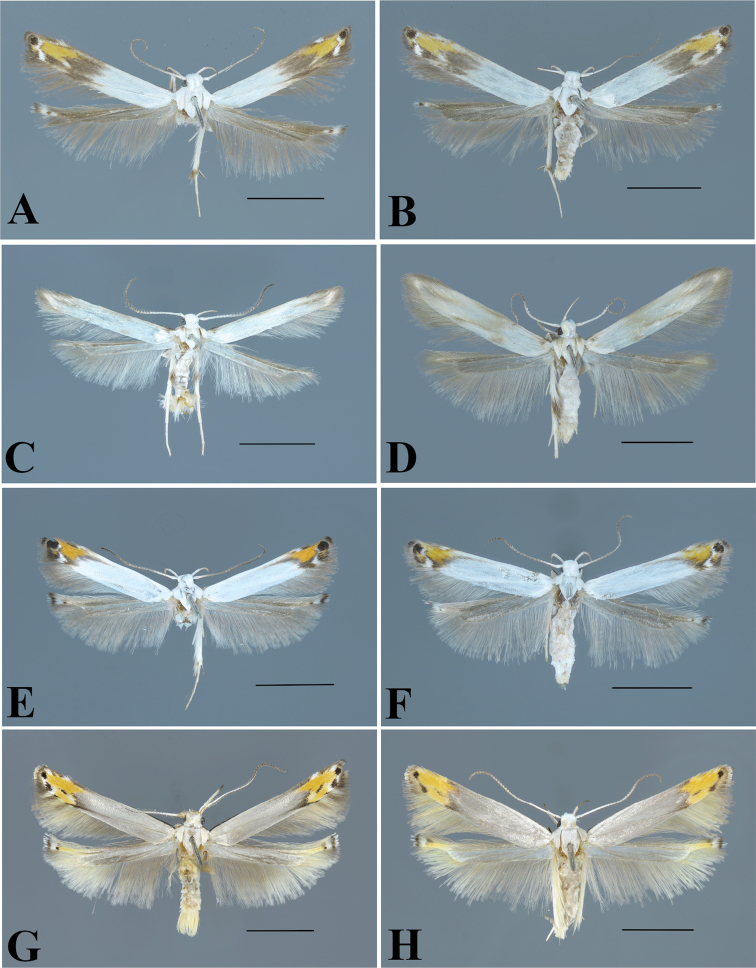
Adults of *Thiotricha* spp. **A***T. biformis*, male **B** ditto, female **C***T. angustella* comb. nov., male **D** ditto, female **E***T. venustalis* comb. nov., male **F** ditto, female **G***T. chujaensis* (Park, 2016) comb. nov., male **H** ditto, female. Scale bars: 2 mm.

### Thiotricha angustella

Taxon classificationAnimaliaLepidopteraGelechiidae

(Omelko, 1984) comb. nov.

4383E1E7-B730-5F67-A5F5-93683DED7ACE

Figs 4C, D , 7B , 8C, D , 9B , 10B

Cnaphostola angustella Omelko, 1984: 32; Omelko 1999: 183; Oku 2003: 65; Park and Ponomarenko 2007: 45; Ueda 2013: 298.

#### Material examined.

Japan – **Hokkaido** [Hokkaido] • 1♀; Yamato, Erimo; 15 Jul. 2002; H. Kogi leg.; Host: *Quercus dentata*; TO • 1♂; same locality and collector; 19 Jul. 2003; Host: *Quercus dentata* • 1♂; Syoya, Erimo; 30 Jun. 2002; H. Kogi leg. • 1♂; Tomikawa, Monbetu; 21 Jul. 2004; H. Kogi leg.; Host: *Quercus dentata*; TO • 3♀♀; same locality and collector; 23–25 Jul. 2006; 6 vii 2007 • 2♀♀; Higasihayakita, Hayakita; 20 Jul. 2005; H. Kogi leg. • 1♂; Tiyosibetu, Hamamasu; 13 Jul. 2002; H. Kogi leg. • 1♂; Katsuranosawa, Atuta; 21 Jul. 2002; H. Kogi leg. • 1♂; Uenai, Tomakomai; 26 Jul. 2002; H. Kogi leg. – **Honshu** [Iwate] • 1♀; Iwayama, Morioka; 2 Jul. 2009; T. Oku leg.; Host: *Quercus mongolica*; 13 Jul. 2009 em.; TO. – **Honshu** [Nagano] • 1♀; Kojiro, Tenryu-mura; N. Hirano leg.; 5 Jul. 2008; gen. slide no. KM-118 • 1♂; same locality and collector; 28 Aug. 2009; gen slide no. KM-106. – **Honshu** [Aichi] • 1♂; Asahikogen, Asahi-cho; 7 Jul. 2001; T. Mano leg.; OPU. – **Honshu** [Mie] • 1♂; Hijiki 250m, Ueno-city; 27 Jun. 1997; T. Mano leg.; gen. slide no. KM-1; OPU • 1♂; Hudodani, Miyama-tyo; 30 Jun. 2001; T. Mano leg.; gen. slide no. KM-96; OPU. – **Honshu** [Kyoto] • 1♂; Mt.Ponpon; 1 Jul. 2000; N. H. Ahn leg.; gen. slide no. KM-2; OPU. – **Honshu** [Nara] • 1♀; Wasamatayama; 9–10 Aug. 1989; S. Moriuti leg.; gen. slide no. KM-51; OPU.

#### Diagnosis.

The forewing is white with pale brown in the distal part, without an apical black spot. The anellus lobe of the male genitalia is a pear-shaped lobe basally, long, strongly sclerotized, and spine-like apically; the valva is narrow and elongate. The apophysis posterioris of the female genitalia is approx. two times of the length of papilla analis and approx. three times longer than the apophysis anterioris; the signum is long, narrow, and arch-shaped.

#### Description.

**Male** (Figs 4C, 7B). Forewing length 2.7–3.4 mm. Wing expanse 6.0–7.3 mm.

***Head***: shiny creamy white with appressed scales. Antennae filiform, basal segment elongate without pecten and creamy white; flagellum creamy white on dorsal surface before middle, then grayish brown beyond, with extraordinarily long and fine cilia on its ventral surface. Labial palpus white, long, and recurved; first segment shortest, creamy white suffused with brown scales on outer surface; second segment thickened, up to 2.5 times the length of the first and white; third segment nearly as long as second, creamy white evenly on both surfaces, apex sharply acute.

***Thorax***: creamy white. Tegula shiny, creamy white dorsally, ornamented with bronze-brown scales along anterior margin.

***Legs***: white; forefemur, tibia, and tarsus suffused inwardly with brown; hind tibia creamy white, with a row of long, stiff, stout white bristles at approx. the midpoint anteriorly, with dark brown bristles at ca. 1/4 posteriorly on dorsal surface, with white bristles ventrally.

***Forewing***: eleven veins, R_4_ + M_1_ stalked, R_5_ absent, anal vein furcate (Fig. 7B). Forewing ground color shiny creamy white, somewhat rounded and pointed apically, with a brownish hue apically 1/5 of the way beyond costal margin; cilia well-fringed, brown from costal area before apex and brownish white along outer margin to inner base of wing.

***Hindwing***: narrower than forewing, white to whitish brown; cilia well-fringed, white to brownish white; apex produced conspicuously.

***Male genitalia***: (Figs 8C, D, 9B) eighth abdominal sternite triangular, long, slightly broadened at base, and then tapered toward posterior with a blunt tip. Uncus swollen, like a small tubercle, short and fine hairs on its top. Gnathos short and stout, its posterior margin closer to base with a hump-like outgrowth; closer to top, somewhat expanded, with a blunt tip. Tegumen much longer than uncus, with dense hairs at approx. the midpoint of its length on dorsal surface. Anellus lobe quite long and pear-shaped basally, bearing a rather long and strongly sclerotized spine-like process at apex, slightly acute and curved inward. Valva elongate, slender, slightly expanded basally, narrow to 3/4 of its length, moderately dilated, and lobate with fine, dense hairs on its inner surface apically, moderately curved inwardly, exceeded apex of tegumen. Vinculum narrow, bearing a few rather long spines on median process of vinculum. Saccus broad basally and U-shaped. Phallus long, small, and spherical at base, and extended distally.

**Female** (Fig. 4D). Forewing length 3.2–3.4 mm. Wing expanse 7.1–7.5 mm. Similar to male.

***Female genitalia***: (Fig. 10B) papillae anales nearly half the length of the apophysis posterioris, with two lobes and long and short fine setae on its entire surface. Apophysis posterioris as much as three times the length of apophysis anterioris. Ostium opening near posterior margin of 8^th^ sternite. Ductus bursae narrow, nearly equal in length to corpus bursae and moderately sclerotized. Ductus seminalis arising from the posterior third of ductus bursae. Corpus bursae oblong; signum long and a narrow arch shaped at left side wall of posterior end.

#### Distribution.

Japan (Hokkaido, Honshu), Russia, Korea.

#### Host plant.

*Quercus dentata* (Kogi 2008), *Q. mongolica* (Fagaceae) (new host record).

#### Biology.

Kogi (2008) reported that the larvae of *T. angustella* occur in August until the following June and make a portable case with fragments of a host plant leaf. The mature larvae use a larger piece of leaf like a hat. Dr. T. Oku collected a case from *Q. mongolica* in Iwate Prefecture in July.

### Thiotricha venustalis

Taxon classificationAnimaliaLepidopteraGelechiidae

(Omelko, 1984) comb. nov.

C33C34F7-B368-55CA-93AB-379B67EF3C27

Figs 4E, F , 7C , 8E, F , 9C , 10C

Cnaphostola venustalis Omelko, 1984: 32; Oku 2003: 65; Park and Ponomarenko 2007: 45; Ueda 2013: 298.

#### Material examined.

Japan – **Hokkaido** [Hokkaido] • 2♂♂; Katsuranosawa, Uryu-cho, Uryu-gun; 20 Jul. 2018; S. Yagi leg. • 1♂, 1♀; Tomuraushi, Shintoku town; 20 Aug. 2000; H. Kogi leg.; gen. slide no. KM-148 (♂) • 2♂♂; Tokachigaoka, Otofuke-cho; 13 Jul. 2000; T. Hirowatari; N.H. Ahn; Y. Miyamoto; H. Okamoto; K. Yamada leg.; gen. slide no. KM-4, 47; OPU • 1♀; Fukuyama, Hobetu Town; 12 Jul. 2005; H. Kogi leg. • 1♀; Siratukari, Atuta; 21 Apr. 2003 em.; H. Kogi leg. • 1♀; Sibi Isikari; 5 Jul. 2016; H. Kogi leg. • 1♂, same locality, 8 Jul. 2007, H.Kogi; 1♂; Ishikari-hama, Ishikari-shi; 18 Jul. 2018; S. Tomura leg. • 3♂♂,1♀; Oyafunebochi, Oyafune-cho, Ishikari; 18 Jul. 2018; S. Yagi leg. • 1♂; Sinkoh, Isikari; 15 Jun. 2003 em.; Host: *Quercus dentata*; H. Kogi leg.; gen. slide no. KM-142 • 1♀; same locality and collector; 25 Jul. 2005 em; Host: *Quercus crispula*; H. Kogi leg. • 1♂; Moiwa, Tomari; 24 Jul. 2006; H. Kogi leg.; gen. slide no. KM-141 • 1♀; Asari-pass, Otaru; 22 Jul. 2002; H. Kogi leg. • 1♀; Tomakomai shi, Kashiwabara; 21 Jul. 2018; S. Tomura leg. – **Honshu** [Iwate] • 1♂; Dogamori, Morioka; 13 Jul. 1994; N. Doi leg.; TO. – **Honshu** [Chiba] • 1♂; Otake, Narita-shi; 18 Jun. 2016; O. Saito leg. – **Honshu** [Gifu] • 1♀; Oniiwa-onsen, Hiyoshi, Mizumani; 1 Jun. 2017; S. Yagi leg. – **Honshu** [Nagano] • 1♂; Kojiro, Tenryu mura; 5 Jul. 2008; N. Hirano leg.; gen. slide no. KM-108 • 1♀; Reisengoya 2260m, Mt. Norikura, Azumi-vill; 20 Jun. 2001; T. Mano leg.; OPU. – **Honshu** [Aichi] • 1♂; Asahi-highland, Asahi-cho; 13 Jul. 1996; T. Mano leg.; gen. slide no. KM-50; OPU • 1♀; same locality and collector; 7 Jul. 2001; OPU. – **Honshu** [Kyoto] • 1♂; Mt. Ponpon; 1 Jul. 2000; N. H. Ahn leg.; gen. slide no. KM-117; OPU • 2♀♀; same label; gen. slide no. KM-48; OPU – **Honshu** [Osaka] • 1♂; Ikoma, 30 Jun. 1995; S. Kosino leg.; gen. slide no. KM-135; OPU • 1♂; Rokumanji-cho, Higashiosaka-shi; 3 Sep. 2017; H. Shimizu leg. • 1♂, 1♀; Aokaiyama (Toyono-tyo); 7 Jul. 1999; T. Saito leg.; gen. slide no. KM-5(♂), KM-49(♀); OPU • 1♀; same label; 21 Jun. 1999; gen. slide no. KM-52; OPU • 1♀; Iwawaki; 28 Jun. 1952; T. Kodama leg.; OPU • 1♀; Izumi-katuragisan (Kisiwada-si); 17 Aug. 2004; T. Saito leg.; gen. slide no. KM-115; OPU. – **Honshu** [Hiroshima] • 1♂,2♀♀; Yoshiwa, Hatsukaichi-shi; 15–16 Jul. 2017; S. Tomura leg. – **Kyushu** [Fukuoka] • 1♂; Nokonoshima, Fukuoka; 3 Jun. 2013; S. Yagi leg.; gen. slide no. KM-101 • 1♂; Hikosan, Soeda-machi; 1 Jul. 2016; S. Yagi leg.; gen. slide no. KM-13 • 1♂; same label; gen. slide no. KM-28 • 1♂; same label; gen. slide no. KM-97. – **Kyushu** [Kumamoto] • 1♂; Taziri Ubuyama; 14 Jul. 2013; S. Yagi leg.; gen. slide no. SY-1.

#### Diagnosis.

The forewing is white with a small orange patch and a black rounded spot apically. The anellus lobe of the male genitalia is narrow, slender, and longer than the valva, with a sharp and thorn-like spine at the apex, which is a unique characteristic of this species. The apopyhsis posterioris of the female genitalia is ca. 1/3 of the length of papilla analis and approx. half the length of the apophysis anterioris; the signum is absent in the corpus bursae.

#### Description.

**Male** (Figs 4E, 7C). Forewing length 2.6–3.6 mm. Wingspan 5.5–7.6 mm.

***Head***: shiny, creamy white with appressed scales. Antennae filiform, basal segment elongate and white; flagellum whitish brown on dorsal surface before middle, then entirely grayish brown beyond, with rather long and fine cilia ventrally. Labial palpus white, long, and recurved; first segment shortest, creamy white; second segment thickened, as much as 2.5 times the length of the first, covered evenly with creamy white scales; third segment as long as second, creamy white, apex sharply acute.

***Thorax and tegula***: creamy white.

***Legs***: white; forefemur, tibia, and tarsus suffused inwardly with dark brown, white on outer surface; mid legs entirely white; hind femur and tibia white, dispersed inwardly with brown; with compact ventral and dorsal rows of long, stiff, stout white bristles; all tarsal segments grayish brown.

***Forewing***: eleven veins, R_3_ + R_4_ stalked, M_1_ separate, R_5_ absent, anal vein furcate (Fig. 7C). Forewing ground color creamy white to white to ca. 3/4 of the way from base, large orange patch of more or less inverted triangular shape along costal margin, extending to apex of wing; large round black spot apically, bordered by a white line with an orange patch; outer margin blackish brown; small brown scales below apical point; diffused brown scales below orange patch at tornal area; cilia well-fringed, brown-white on costal margin before apex, dark brown with fuscous median band from apex to termen, grayish brown through inner base of the wing.

***Hindwing***: narrower than forewing, brown with tiny dark brown apical dot; fringe around apex darker in color, long, and brown, cilia well-fringed on inner region of hind wing.

***Male genitalia***: (Figs 8E, F, 9C) eight abdominal sternite mucronate, short and wide surfaces, anterior 2/3 broadly concave, and then narrow beyond, slightly sharpened basally. Uncus swollen and rounded with short, fine setae on apex. Gnathos sickle-shaped, short and stout, flattened at base posteriorly and then slightly curved toward apex. Tegumen long, nearly three times length of uncus, with dense hairs at approximately its midpoint on dorsal surface. Anellus lobe, a pair of slender processes, slightly longer than valva, slightly broadened and elbowed basally, then narrowly elongate and slightly swollen 1/4 of the way before apex, with rather long and weakly sclerotized thorn-like spine at tip. Valva enlarged at base, with long fine setae on rim of inner surface, gradually narrowing from base to 2/3 of length, slightly curved inwardly, a few setae at corner of anterior margin, rhomboid with a blunt tip apically, short and fine setae on inner and outer surfaces. Vinculum slightly narrow and enlarged surface, a few setae on its median process. Saccus broad basally, somewhat inflate and convex. Phallus large and clavate basally, narrowly elongate in distal half.

**Female** (Fig. 4F). Forewing length 2.6–3.5 mm. Wing expanse 6.2–7.2 mm. Similar to male.

***Female genitalia***: (Fig. 10C) papilla analis bilobed, ca. twice length of apophysis anterioris, with long and short fine setae on its entire surface. Apophyses short but apophysis anterioris ca. two times longer than apophysis posterioris. Ostium opening near anterior margin of 8^th^ sternite. Ductus bursae nearly as long as corpus bursae and weakly sclerotized. Corpus bursae slightly oblong; signum absent.

#### Distribution.

Japan (Hokkaido, Honshu, Kyushu), Russia, Korea.

#### Host plant.

*Quercus dentata* (Kogi 2004), *Q. crispula* (Fagaceae) (new host record).

#### Biology.

Kogi (2004) reported that adults of this species fly in July in Hokkaido and larvae live in portable cases in summer until the following spring. In late fall, the larvae move to the underside of twigs of the host plant for hibernation. The next spring, the larvae create triangular cases like hats with fragments of host plant leaves and skeletonize the leaves.

### Thiotricha chujaensis

Taxon classificationAnimaliaLepidopteraGelechiidae

(Park, 2016) comb. nov.

A1D2408E-CD4B-5B80-B430-12FE5C330042

Figs 4G, H , 7D , 8G, H , 9D , 10D , 11A–I , 13A–C , 14A–C

Cnaphostola chujaensis : Park and Kim 2016: 172, fig. 1.

#### Material examined.

Japan – **Honshu** [Chiba] • 1♀; Kayano, Orikisawa, Kimitsu-shi; 4 Sep. 2013; O. Saito leg. • 1♂; Otake, Narita-shi; 23 Jul. 2016; O. Saito leg. • 1♀; same locality and collector; 20 Aug. 2016 • 1♂; same locality and collector; 3 Jun. 2017 • 1♀; same locality and collector; 9 Sep. 2017 • 1♀; same locality and collector; 7 Oct. 2017. – **Honshu** [Ishikawa] • 1♀; Hodatsushimizu cho, Shikinami; 16 Jun. 2018; S. Tomura leg. – **Honshu** [Nagano] • 2♀♀; Kojiro, Tenryu-mura; 19 Jun. 2009; 28 Aug. 2009; N. Hirano leg. • 1♂; Hiraoka, Tenryu-mura; 16 Jun. 2007; N. Hirano leg. [Gifu] • 1♀; Yamagata-gun, Miyama-cho, Iodo; 17 Jun. 1994; T. Mano leg.; gen. slide no. KM-140; OPU. – **Honshu** [Shizuoka] • 1♂; Oonogi Umegasima, Shizuoka-city; 25 Aug. 2001; T. Mano leg.; gen. slide no. KM-32; OPU • 1♀; Shizuoka city, Hirano, Abe river; 23 Aug. 1997; T. Mano leg.; OPU • 1♀; Hirano, Shizuoka city; 26 Aug. 1995; T. Mano leg.; OPU • 1♀; Konya Spa, Shizuoka city; 27 Jul. 2002; T. Oku leg.; TO. – **Honshu** [Aichi] • 3♂♂; Zaikaji-temple, Toyokawa city; 4 Jun. 1994; T. Mano leg.; gen. slide no. KM-18; OPU • 1♂; Somasaka Pass, Toyokawa city; 21 May. 1992; T. Mano leg.; gen. slide no. KM-20; OPU • 2♀♀; same label; gen. slide no. KM-79; OPU • 1♀; Matsumine-cho, Toyota city; 13 Jun. 2001; T. Mano leg.; gen. slide no. KM-77; OPU. – **Honshu** [Mie] • 1♂; Yamadano (Hakusan-tyo); 18 Apr. 2001; Host: *Mallotus japonica* (“shinme”[=a shoot]); 30 Apr. 2001 em.; T. Saito leg.; OPU • 1♂,1♀; same locality and collector; 23 Apr. 2002; gen. slide no. KM-12(♂); OPU • 6♂♂, 2♀♀; same label; 23 Apr. 2002; Host: *Mallotus japonica* (“shinme”[=a shoot]); 30 Apr.–6 May. 2002 em.; gen. slide no. KM-65; OPU • 1♀; Obara-ishiki, Hokusei-cyo; 7 Sep. 1997; T. Mano leg.; OPU • 1♀; Mikuni valley alt 430 m, Hujiwara-cho; 27 Aug. 1998; T. Mano leg; OPU • 1♂; Hijiki 250m, Ueno City; 12 Sep. 1997; T. Mano leg.; gen. slide no. KM-31; OPU • 1♀; Inabe-gun, Fujiwara-cho, Shinodachi; 29 Aug. 1994; T. Mano leg.; OPU. – **Honshu** [Shiga] • 1♂; Makino, Takashima-shi; 13 May. 2015; H. Shimizu leg. – **Honshu** [Kyoto] • 1♂; Mt. Ponpon; 1 Jul. 2000; N. H. Ahn leg.; gen. slide no. KM-34; OPU • 1♂, 1♀; Yamanouchi-cho, Seikanji, Higashiyama-ku, Kyoto-shi; 1 Jun. 2013; H. Shimizu leg. • 4♀♀; Kanmurijima I., Miyazu-shi; 27–28 Sep. 1982; M. Sasakawa; Y. Yoshiyasu; N. Nishida & T. Kamura leg.; gen. slide no. KM-63; OPU. – **Honshu** [Nara] • 1♀; Kasugayama (Nara-si); 26 Aug. 2000; T. Saito leg.; OPU • 1♂; Mt. Takatoriyama (Takatori) ca 580 m; 25 Aug. 1993; Kadohara leg.; OPU • 1♀; Katuragi-shi, Taima; 7 Aug. 2009; H. Shimizu leg. • 1♀; Sannoko, Kawakami village; 18 Jun. 1991; T. Ueda leg.; OPU • 1♂; Kawakami village, Kitamata; 15 Jun. 1993; T. Ueda leg.; gen. slide no. KM-30; OPU. – **Honshu** [Hyogo] • 1♂; Tamida (Inagawa-tyo); 29 Apr. 2002; T. Saito leg.; Host: *Mallotus japonica*; 3 May. 2003 em.; gen. slide no. KM-33; OPU. – **Honshu** [Osaka] • 1♀; Aokaiyama (Toyono-tyo); 10 Jun. 1999; T. Saito leg.; gen. slide no. KM-62; OPU • 1♀; Minou River, Minou City; 5 Aug. 1991; M. Aoyagi & T. Ueda leg.; OPU • 1♂; Higasiosaska-shi, Rokumanji-cho; 23 May. 2015; H. Shimizu leg. • 1♂; same locality and collector; 18 May. 2017 • 1♂; Yao-shi, Kodachi, Jyusan-toge, Fumin no mori; 16 Jul. 2011; H. Shimizu leg. • 1♀; same locality and collector; 17 May. 2016 • 1♀; Hatigamine; 4 Jun. 1993; S. Koshino leg.; gen. slide no. KM-66; OPU • 1♂,1♀; same locality and collector; 15 Jul. 1993; gen. slide no. KM-19 (♂), 126(♀); OPU • 1♀; Minamikawachi-gun, Mizukoshi-toge, Mt. Kongo; 7 Jun. 2015; H. Shimizu leg. • 2♀♀; Tondabayasi Dakeyama ca. 260 m; 4 Sep. 1992; Kadohara leg.; OPU • 1♀; Kawachinagano city, Iwawaki temple; 3 May. 2001; T. Saito leg.; Host: *Mallotus japonica*; 20 May. 2001 em.; OPU • 1♀; Kawati, Iwawakisan; 29 May. 1954; T. Yasuda leg.; OPU • 1♀; Kawakubo; 19 Sep. 1995; S. Kosino leg.; gen. slide no. KM- 72; OPU • 1♀; Tottori (Hannan); 18 May. 1997; S. Kosino leg.; OPU • 3♀♀; Izumi-katuragisan (Kisiwada-si); 17 Jul. 2004; T. Saito leg.; gen. slide no. KM-42; KM-64; OPU • 1♀; same locality and collector; 21 Jul. 2004; OPU • 1♀; Kyosi, 21 May. 1998; S. Kosino leg.; gen. slide no. KM-128; OPU. – **Honshu** [Wakayama] • 1♀; Wakayama-shi, Nougawa; 11 Jun. 2007; M. Murase leg.; OPU • 1♂, 1♀; Wakayama-shi, Yata; 3 Jul. 2001; T. Hirowatari; B. W. Lee; N. H. Ahn; Y. Miyamoto & K. Yamada leg.; gen. slide no. KM-35(♂),60(♀); OPU. – **Shikoku** [Ehime] • 1♀; Matsuyama; 16 May 1957; M. Okada leg.; gen. slide no, KM-71; OPU. – **Kyushu** [Fukuoka] • 1♂; Orio; 4 Jul. 1958; T. Kawamura leg.; gen. slide no. KM-21 • 2♀♀; Orio; 24 Jun. 1959; T. Kawamura leg.; gen. slide no. KM-78 • 1♂; Kasii, Fukuoka City; 25 May. 1958; K. Yano leg.; gen. slide no. KM-37 • 1♂; Aburayama, Fukuoka; 24 May. 1959; T. Kawarabata leg.; gen. slide no. KM-26 • 1♂; Atago, Nishi-ku, Fukuoka shi; 26 May. 2018 larva; Host: *Mallotus japonica*; 9 Jun. 2018 em.; S. Yagi leg. • 1♀; same locality, collector and host; 9 Jun. 2018 larva; 9 Jul. 2018 em • 2♂♂; Ito campus, Nishi-ku; 26 May. 2017; K.M.M.Kyaw; gen. slide no. KM-43, 44 • 1♂; Mt. Hikosan; 18 Jun.1962; H. Kuroko leg.; OPU • 1♀; same locality and collector; 1 Sep. 1953; OPU • 1♀; same locality; 20 Aug. 2013; S. Yagi leg.; gen. slide no. KM-70 • 1♀; same locality and collector; 25 Jul. 2014; LT; gen. slide no. KM-69 • 1♀; same locality; 31 Jul. 2014; LT; T. Hirowatari & S. Yagi leg. • 1♂; same locality; 4 Sep. 2014; LT, S. Yagi leg.; gen. slide no. KM-15 • 1♂, 4♀♀; same locality and collector; 27 Jul. 2015; LT; gen. slide no. KM-17(♂), 73(♀), 75(♀) • 1♂; same locality and collector; 13 Aug. 2016; LT; gen. slide no. KM-36. – **Kyushu** [Saga] • 1♂; Hokuzan, Saga-shi; 3 Jun. 2017; S. Tomura leg. – **Kyushu** [Kagoshima] • 1♀; Sata; 18 May. 1952; T. Kodama leg.; OPU. – **Ryukyus** [Kagoshima] • 1♀; Nakama, Yakusima Is.; 20 Sep. 1978; S. Moriuti leg.; OPU • 1♂; Okawa Rindo, Kurio, Yakushima, 120m; 21 Jun. 2017; S. Yagi leg. • 2♂♂, 2♀♀; Hatsuno, Amamioshima Is.; 3–5 Apr. 1996; T. Ueda leg.; gen. slide no. TU-751(♂), KM-14(♂), 68(♀); OPU • 1♀; Mt. Yuwan-dake (Lower), Uken-son vill.; 18 May. 2015; S. Sameshima leg.; gen. slide no. KM-57; KGU • 1♀; Akatsuchiyama, Yuwan, Uken-son; 20 Aug. 2014; S. Sameshima leg.; KGU • 1♀; same locality; 6 Jul. 2016; LT 245m; S. Yagi leg.; gen. slide no. KM-83 • 1♂, 3♀♀; Chuorindo, Amamioshima Is.; 6 Apr. 1996; T. Ueda leg.; gen. slide no. KM-25(♂), 67(♀); OPU • 1♀; Nishinakama, Amamioshima Is.; 6 Jun. 1996; T. Ueda leg.; OPU • 1♂; Fureainomori, Uken vill.; 25 Sep. 2002; gen. slide no. KM-27; Host: *Mallotus japonica*; OPU • 1♂; Mt. Yui-dake, Setouchi-cho, Amamioshima Is.; 30 Jun. 2006; U. Jinbo leg.; NSMT-I-L-35780; • 1♀; Tokuno-shima, San Tokunoshima; 9 Jul. 2016; LT 230 m; S. Yagi leg.; gen. slide no. KM-59 • 1♀; Tokuno-shima, Fugusuku; 11 Jul. 2016; S. Yagi leg. – **Ryukyus** [Okinawa] • 1♂; Mt. Terukubi 330 m; Benoki, Kynigami-son; 5 Aug. 2015; L.T; S. Yagi leg.; gen. slide no. KM-29 • 1♀; Yona, Kunigami vill.; 15–18 May. 1998; T. Ueda leg.; OPU • 1♀; Mt. Fuenchijisan, Kunigami vill.; 8 Jun. 1997; T. Ueda leg.; gen. slide no. KM-74. • 1♀; Hentona; 9 Aug. 2016; LT 60 m; S. Yagi leg. • 1♀; Uka, Kunigami-son, Kunigami-gun; 31 May. 2015; 250m LT; S. Yagi leg. • 1♀; Banna Park, Ishigaki Is.; 31 Mar. 2002; B. W. Lee leg.; OPU; gen. slide no. KM-87 • 1♀; Mt Omoto, Ishigaki Is.; 11 Nov. 2003; Host: *Mallotus japonica*; 18 Nov. 2003; S. Shimizu leg.; OPU; gen slide no. KM-76.

#### Diagnosis.

This species is similar to *T. biformis*, which is known in the Russian Far East and Japan but can be distinguished by markings in the distal yellow zone of the forewing with a distinct black streak below the middle of the yellow zone. The male genitalia is similar to those of *T. epiclista* Meyrick, 1908 described from Khasi Hills, India, but can be distinguished by the basally broadened valva and the presence of a thumb-like basal process bearing numerous setae, the lack of a pre-apical spine on the costal process of the valva, and the slender posterior part of the phallus.

#### Description.

**Male** (Figs 4G, 7D). Forewing length 2.7–4.3 mm. Wing expanse 5.8–8.8 mm.

***Male genitalia***: (Figs 8G, H, 9D).

**Female** (Fig. 4H). Forewing length 3.0–4.4 mm. Wing expanse 5.9–9.1 mm. Similar to male.

***Female genitalia***: (Fig. 10D) see Park and Kim (2016) for detailed descriptions of the adults and genitalia. In the present study, intraspecific variation observed was the presence or absence of a blackish streak on the yellowish zone of the forewing markings in all examined specimens. Additionally, in contrast, the biggest size of the adult moth of Japanese specimens (wing span 8.8 mm) is quite smaller than that of Korean specimens (wing span 12 mm in Park and Kim’s description). In the female genitalia also, Park and Kim described the shape of the signum forming as an elongate plate with dense spicules and located at posterior end; however, that of the Japanese specimens is developing as a pentagonal shape at the posterior end in our observation.

#### Distribution.

Japan (Honshu, Shikoku, Kyushu, Ryukyus), Korea.

#### Host plant.

*Mallotus japonica* (Euphorbiaceae).

#### Biology

(Fig. 11). Although the host plant of *T. chujaensis* is unknown, we found that some specimens of this species preserved in OPU were labeled as having been reared on *Mallotus japonica* (Euphorbiaceae). In the present study, we confirmed that the larvae feed on this plant in the field. The larvae also make portable cases with flower buds or attack the leaf bud (young shoot) of the host plant. The larva uses the flower buds at flowering time to make a case from the plant. After making the portable case, the larva moves from one place to another and attaches the case to the lower surface of the leaf for pupation (Fig. 11C). When there is an early leaf bud (young shoot) on the host plant, the larva penetrates the petiole of the young, newly emerged leaf and feeds inside (Fig. 11E–G). There is no external injury during the feeding period until the leaf is fully grown. Before pupation, the larva cuts the petiole and makes a portable case. After that, it fixes the portable case sideways. Pupation also takes place inside the cases and pupal exuvia is left after the adult emerges.

#### Pupa

(Figs 13A–C, 14A–D). Length ca. 3.2 mm, cylindrical. Color yellowish brown; dark brown before emergence. Vertex armed with many minute spines. Prothorax with a pair of not truly triangular projections on anterolateral corners of tergite. Antenna reaching to posterior margin of 6^th^ abdominal segment. Forewing reaching the mid-way of 6^th^ abdominal segment. Forelegs extending to 3^rd^ abdominal segment; midlegs extending to mid-way of 5^th^ abdominal segment; hindlegs also extending to near posterior margin of 7^th^ abdominal segment. Abdominal segment 7^th^ armed with a row of distinct tergal spines directed posteriorly on anterior margin and indistinct short tergal spines on caudal margin. Seventh abdominal sternite with a pair of oval pads also armed with a row of spines directed anteriorly. Tenth abdominal segment with a pair of triangular projections at middle, no true cremaster present.

#### Remarks.

This species was described by Park (in Park and Kim 2016) from Chuja Islands which is one of the largest islands among 42 islands in the Jeju Strait, approximately halfway between Jeju Island and the southern coast of the Korean Peninsula. In the present study, it was found that this species is very common and widely distributed in southern Japan.

### Thiotricha elaeocarpiella

Taxon classificationAnimaliaLepidopteraGelechiidae

Kyaw, Yagi & Hirowatari sp. nov.

EB01F302-B41C-5551-94E3-114082D4FF0F

http://zoobank.org/F05504CD-56BC-4318-855B-5B3640B9D1D2

Figs 5A, B , 6A, B , 7E , 8I, J , 9E , 10E , 12A–I , 13E, F , 14E, F

Cnaphostola sp. 2: Oku et al. 2018: 30, fig. 45.

#### Type material.

***Holotype***: Japan – **Kyushu** • 1 ♂, Fukuoka Pref., Kyushu Univ. Ito Campus, Nishi-ku; 7 Aug. 2017; S. Yagi, T. Hirowatari, K. M. M. Kyaw & C. Tsuji leg.; case on *Rhaphiolepis indica* (case made from flower bud of *Elaeocarpus zollingeri*); 19 Aug. 2017 em.; gen. slide no. KM-88; in ELKU.

***Paratypes***: Japan – **Kyushu** [Fukuoka] • 1♂; same locality and collectors as holotype; 26 May. 2017; portable case on *Rhaphiolepis indica*; 17 Jul. 2017 em.; gen. slide no. KM-40 • 3♂♂; same locality and collectors as holotype; 31 Jul. 2017; Host: *Rhaphiolepis indica*; 7 Aug. 2017 em.; gen. slide no. KM-104,105 • 2♂♂; same locality and collector as holotype; 22 Jul. 2017; Host: *Rhaphiolepis indica*; 31 Aug. 2017 em. • 1♀; same locality; 22 Jul. 2017; Host: *Rhaphiolepis indica*; 27 Aug. 2017; K.M.M.Kyaw leg.; gen. slide no. KM-132. – **Kyushu** [Kagoshima] • 1♀; Satahetsuka (L), Minamiousumi Town; 9–10 Jul. 2011; T. Terada leg. (KGU). – **Ryukyus** [Kagoshima] • 1♂, 1♀; Amami-Oshima Is., Mt. Yuwan-dake, Uken; 17 Aug. 2012; S. Sameshima leg.; gen. slide no. KM-23(♂), 56(♀); KGU • 1♀; same locality; 4 May. 2013; K. Tsuda leg.; KGU • 1♀; same locality; 4 Aug. 2014; S. Sameshima leg.; KGU • 1♂; same locality; 5 May. 2015; S. Sameshima leg. (KGU); gen slide no. KM-24 • 4♀♀; Akatsuchiyama, Yuwan, Uken-son, 245m; 6 Jul. 2016; LT; S. Yagi leg.; KM-82; 116; 125. – **Ryukyus** [Okinawa] • 1♂; Okinawa ken, Higashi son Kunigami, Takae; T. Hirowatari, S. Yagi, K.M.M.Kyaw leg. • 1♀; Kenmin no-mori, Afuso; 11 Aug. 2017 (larva); Host: *Elaeocarpus zollingeri*; 29 Aug. 2017 em.; same collectors; gen. slide no. KM-89.

#### Diagnosis.

At a glance, the external features are similar to those of *T. chujaensis* (Park, 2016) comb. nov. but it can be distinguished by wing markings in the distal yellow zone of the forewing, which lacks a distinct blackish streak below the middle of the yellow zone and features grayish scales at the costal margin before the apex and the area beyond the tornus. Additionally, it can easily be distinguished based on the male genitalia; the uncus is more rounded apically; the gnathos is U-shaped and acute apically; the valva is narrowly elongate with a sharped pre-apical process ca. 1/4 of the way along its length and the vinculum lacks thumb-like lobes posteriorly; the saccus has a rounded base. The shape of the phallus is also different. However, the male genitalia are similar to those of *Thiotricha clidias* Meyrick, 1918, which was described from Khasi Hills, India, although they differ in the shape of the phallus. In *T. clidias* Meyrick, 1918, the phallus is rounded basally, abruptly sinuate and slender in distally but as a cucurbit-shaped in *T. elaeocarpiella* sp. nov.

#### Description.

**Figure 5.**
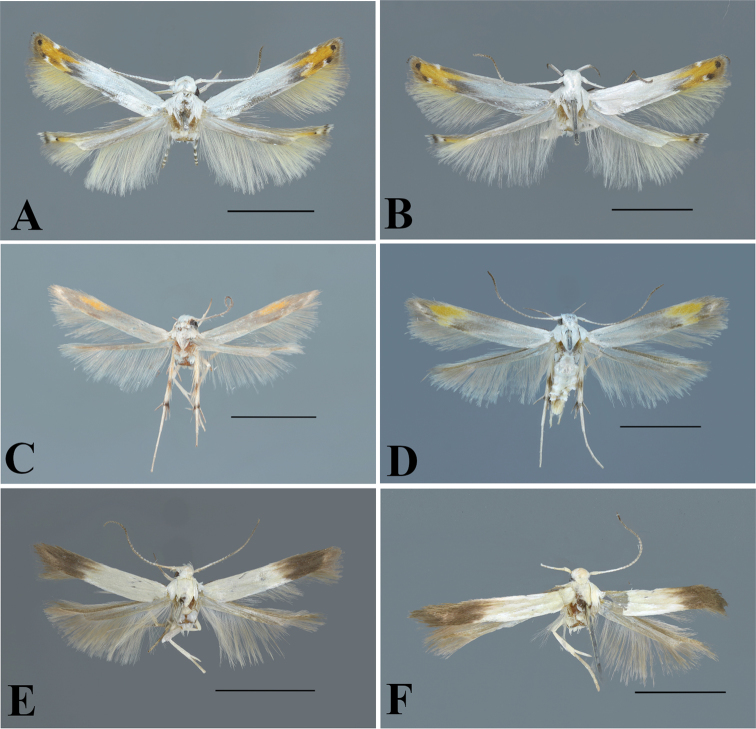
Adults of *Thiotricha* spp. **A***T. elaeocarpiella* sp. nov., male (holotype) **B** ditto, female (paratype) **C***T. flavitermina* sp. nov., male (holotype) **D** ditto, female (paratype) **E** ditto, male (paratype) **F** ditto, female (paratype). Scale bars: 2 mm.

**Figure 6.**
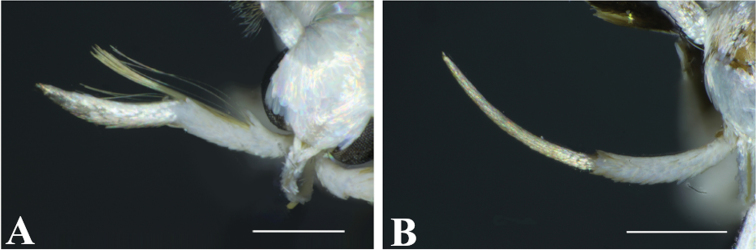
Labial palps of *Thiotricha elaeocarpiella* sp. nov. **A** male with hair pencils, paratype **B** female without hair pencils., paratype. Scale bars: 0.4 mm.

**Figure 7.**
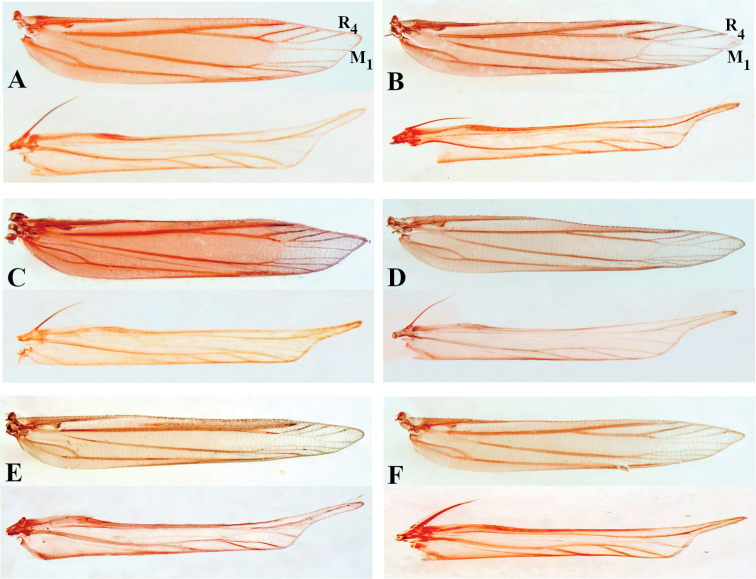
Wing venation of *Thiotricha* spp., male. **A***T. biformis***B***T. angustella* comb. nov. **C***T. venustalis* comb. nov. **D***T. chujaensis* (Park, 2016) comb. nov. **E***T. elaeocarpiella* sp. nov., paratype **F***T. flavitermina* sp. nov., paratype.

**Male** (Figs 5A, 6A, 7E) Forewing length 2.9 mm in holotype, 2.6–3.4 mm in paratypes. Wing expanse 6.4 mm in holotype, 5.2–7.1 mm in paratypes.

***Head***: shiny creamy white with appressed scales. Antennae filiform, basal segment elongate and creamy white, sparsely speckled with brown scales; flagellum grayish white on dorsal surface before midpoint, then brownish gray beyond on its dorsal and ventral surfaces, with extraordinarily long and fine cilia ventrally. Labial palpus white, moderately long and recurved; first segment approximately half the length of the second, with blackish gray scales on lateral surface; second segment as much as 1.5 times the length of the first, creamy white throughout on outer surface; bundle of hair pencils arising from apex of first and second segment, appressed on dorsal surface to near the end of the third segment; third segment as thick as second, with blackish gray scales medially on lower surface ventrally, shiny creamy white evenly on both surfaces, apex sharply acute (Fig. 6A).

***Thorax***: creamy white. Tegula shiny, creamy white dorsally, ornamented with blackish gray scales along anterior margin.

***Legs***: white; forefemur, tibia, and tarsus suffused inwardly with blackish brown; scattered with white scales on outer surface; mid femur entirely white; mid tibia and tarsus white but slightly speckled with blackish brown scales on outer surface; hind femur white; hind tibia creamy white, with a row of long, stiff, stout, creamy white bristles above and below, suffused with a small blackish gray scale on lateral outer surface posteriorly; first tarsal segment entirely blackish gray; second and third segment white with blackish gray apical ring; last two segments white.

***Forewing***: eleven veins, R_4_ + M_1_ stalked, R_5_ absent, anal vein furcate (Fig. 7E). Forewing ground color shiny grayish white to white to ca. 3/4 of the way from base; distinct orange zone in distal 1/4, deeply concave along costal margin; costal margin and area beyond tornus grayish colored; small black spot at apex, narrowly connected to another black spot in tornus; cilia before apex to tornus brown, creamy yellow from tornus to inner base of wing.

***Hindwing***: narrower than forewing, creamy white to grayish white, with pale orange apical zone; apex sharply produced, with small apical black spot; cilia well-fringed to base, fringe around apex creamy white, with broad, dark brown median band.

**Figure 8.**
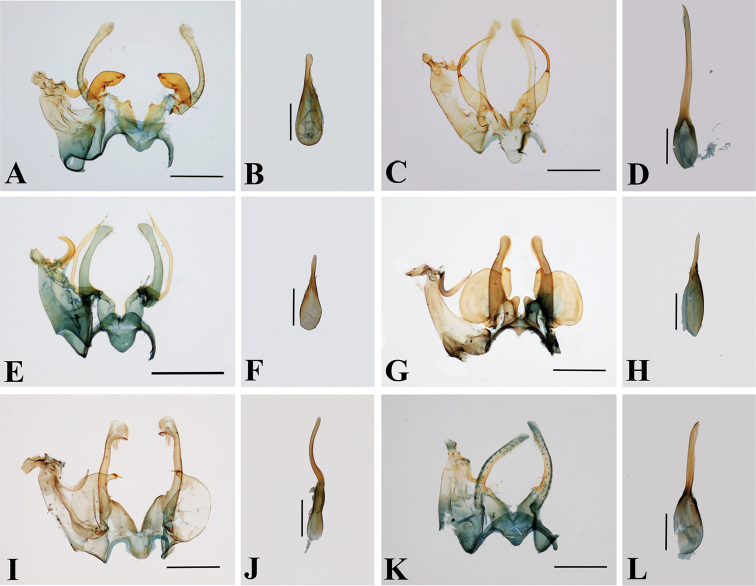
Male genitalia of *Thiotricha* spp. **A, B***T. biformis***A** male genitalia, gen. slide no. KM-143 **B** phallus **C, D***T. angustella* comb. nov. **C** male genitalia, gen. slide no. KM-106 **D** phallus **E, F***T. venustalis* comb. nov. **E** male genitalia, gen. slide no. KM-1 **F** phallus **G, H***T. chujaensis* (Park, 2016) comb. nov. **G** male genitalia, gen. slide no. KM-43 **H** phallus **I, J***T. elaeocarpiella* sp. nov., holotype **I** male genitalia, gen. slide no. KM-88 **J** phallus **K, L***T. flavitermina* sp. nov., holotype **K** male genitalia, gen. slide no. KM-100 **L** phallus. Scale bars: 0.4 mm (genitalia), 0.2 mm (phallus).

**Figure 9.**
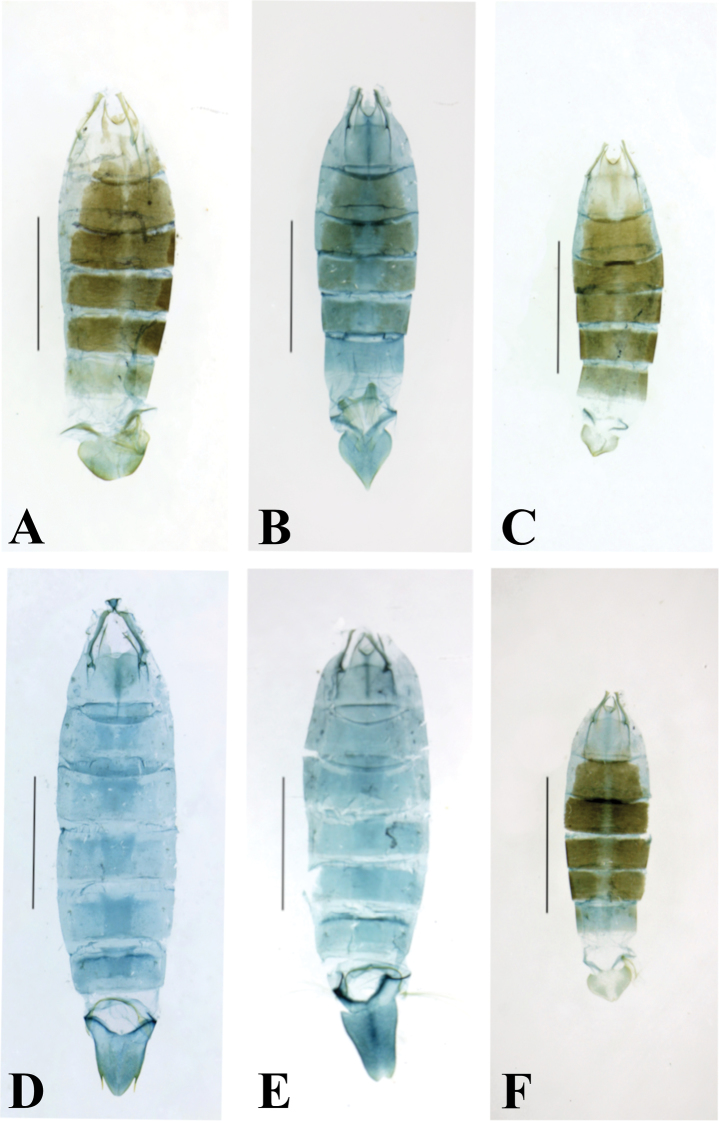
Abdominal segments of *Thiotricha* spp. **A***T. biformis*. **B***T. angustella* comb. nov. **C***T. venustalis* comb. nov. **D***T. chujaensis* (Park, 2016) comb. nov. **E***T. elaeocarpiella* sp. nov., paratype **F***T. flavitermina* sp. nov. Scale bars: 1 mm.

***Male genitalia***: (Figs 8I, J, 9E) eighth abdominal sternite more or less triangular, emarginate at the tip, slightly broadened basally with moderately sclerotized margin anteriorly, gradually narrow toward apex. Uncus directed backwards, with broad basal expansion, then narrowly elongate, forming a furrow on lower surface medially, bearing short spines on its lateral margin and abruptly rounded with short and fine setae evenly on its apical dorsal surface. Gnathos U-shaped, stout, strongly bent at basal 1/3, sharply acute apically. Tegumen longer than uncus, slightly concave medially on lateral margins with dense hairs on dorsal surface beyond middle. Anellus lobe, a large process, as much as 1/2 the length of process of valva, ovate membranous pouch at base, with short sclerotized apical spine and short fine setae around apical spine. Valva slender, elongate, broad basally, narrow along 2/3 of length, then dilated apically with dense, long, fine hairs hanging down from its inner surface and developing a sclerotized point, spine-like pre-apical process arising from its base, nearly 1/4 of apex. Vinculum long and slightly narrow, with few rather long setae on its lateral margin. Saccus roundly produced basally. Phallus cucurbit-shaped in basal half, slightly sinuate, slender and recurved upwardly in distal half.

**Female** (Figs 5B, 6B). Forewing length 2.5–3.3 mm. Wing expanse 5.3–7.1 mm. Similar to male but differs as follows: Labial palps of first segment shortest, with creamy white scales, as thick as second segment; second segment as long as third segment and with white scales on lower surface and grayish or grayish brown scales on upper surface; third segment slender and acute with gray to grayish-brown scales on both surfaces (Fig. 2B)

**Figure 10.**
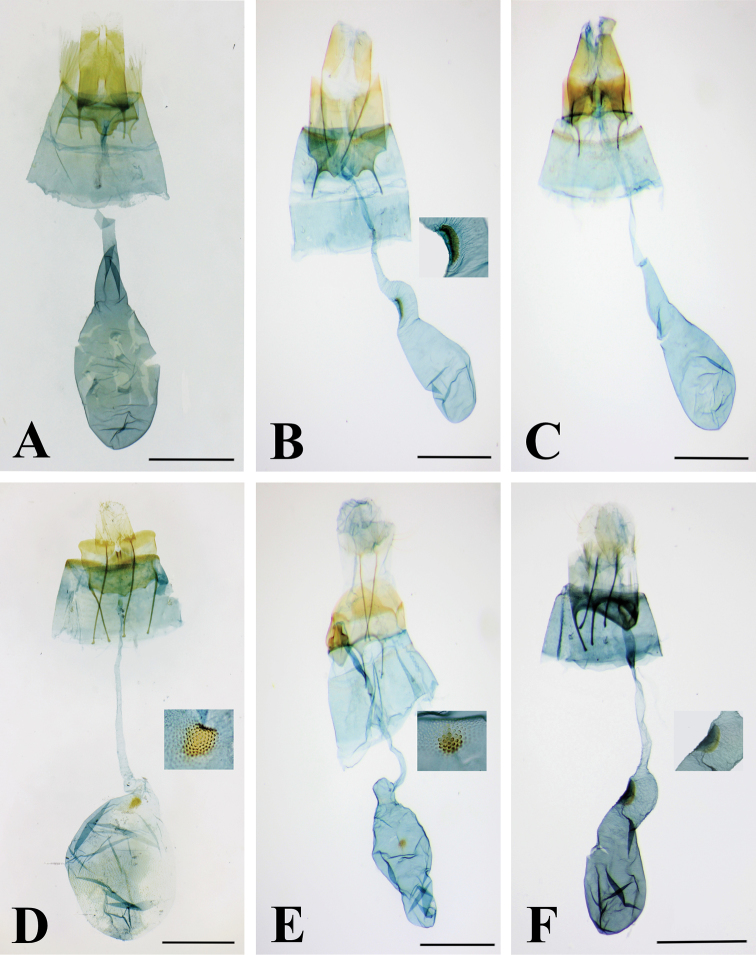
Female genitalia of *Thiotricha* spp. **A***T. biformis*, gen. slide no. KM-145 **B***T. angustella* comb. nov., with close up of signum, gen. slide no. KM-118 **C***T. venustalis* comb. nov., gen. slide no. KM-52 **D***T. chujaensis* (Park, 2016) comb. nov. with close up of signum, gen. slide no. KM-128 **E***T. elaeocarpiella* sp. nov. with close up of signum, paratype, gen. slide no. KM-132 **F***T. flavitermina* sp. nov. with close up of signum, paratype, gen. slide no. KM-55. Scale bars: 0.5 mm.

***Female genitalia***: (Fig. 10E) papillae anales with long and short fine setae on its entire surface. Apophysis posterioris longer than apophysis anterioris; apophysis anterioris ca. 1/3 the length of posterioris. Ductus bursae rather long, narrow, slightly sclerotized along the posterior half of its length. Corpus bursae clavate in shape; signum rounded at center.

#### Distribution.

Japan (Kyushu, Ryukyus).

#### Etymology.

The name refers to its main host plant, *Elaeocarpus zollingeri*.

#### Host plant.

*Elaeocarpus zollingeri* (Elaeocarpaceae), *Rhaphiolepis indica* (Rosaceae).

#### Biology

(Fig. 12). The larva uses the flower bud or the young shoot of its host plant to construct portable cases. When it utilizes a flower bud, at first, the larva penetrates the bud and then lives and feeds within it. After that, it moves from one flower to another by carrying the bud and attaching it to other flower buds to complete its life cycle (Fig. 12D, E). When the larva is ready to pupate, it attaches the case to the underside of a leaf with silk. When it utilizes a young shoot (Fig. 12C), the larva leaves small dot-like traces of feeding after making cases by the shoot. Pupation also takes place inside the portable cases. After completing development, the adult emerges from the case, leaving the pupal exuvia inside.

#### Pupa

(Figs 13D–F, 14A–D). Length ca. 3.2 mm, cylindrical. Color yellowish brown. Vertex armed with many minute spines. Prothorax with a pair of triangular projections on anterolateral corners of tergite. Antenna and forewing reaching to posterior margin of 6^th^ abdominal segment. Forelegs extending to 3^rd^ abdominal segment; midlegs reaching to mid-way of 5^th^ abdominal segment; hindlegs also extending to just beyond the anterior margin of 7^th^ abdominal segment. Seventh abdominal segment armed with a row of distinct tergal spines directed posteriorly on anterior margin and indistinct short tergal spines on caudal margin. Seventh abdominal sternite with a pair of oval pads also armed with a row of spines directed anteriorly. Tenth abdominal segment with a pair of triangular projections at middle, no true cremaster present.

#### Remarks.

Although this new species was found on two different plants in the present study, it may be that *E. zollingeri* is mainly utilized as the host plant and occasional feeding on *R. indica* occurs when individuals happen to come into contact with this plant. See discussion.

### Thiotricha flavitermina

Taxon classificationAnimaliaLepidopteraGelechiidae

Kyaw, Yagi & Hirowatari sp. nov.

2DAD0FF8-0CAB-50E6-B1E5-B91FAD140889

http://zoobank.org/71448E70-4DE6-41EB-A734-F4D5CBEE77F8

Figs 5C–F , 7F , 8K, L , 9F , 10F

Cnaphostola sp. 1: Oku et al. 2018: 29, fig. 44.

#### Type material.

***Holotype***: Japan – **Ryukyu • 1** ♂; Okinawa Pref., Kunigami vill., Ookunirindo; 26, 27 May. 2000; T. Mano leg.; gen. slide no. KM-100; in OPU.

***Paratypes***: Japan – **Ryukyus** [Kagoshima] • 1♂; Tokara Island, Nakanoshima Is, Takao; 15 Nov. 2018; K. Sakagami leg. • 1♂; Mt. Akatuti-yama, Uken-son vill., Amamioshima Is.; 21 May. 2013; S. Sameshima leg.; KGU • 5♀♀; same locality and collector; 8–11 Jun. 2013; KGU • 2♂♂, 1♀; same locality and collector; 25–27 May. 2015 (KGU); gen slide no. KM-3,134(♂), KM-46(♀) • 1♂, 1♀; Nankawa path, Amamioshima Is.; 2 Jun. 2013; S. Sameshima leg.; KM-39(♂); KGU • 1♀; Mt. Yuwan-dake, Uken-son vill.; 19 Jun. 2014; S. Sameshima leg. (KGU) [Okinawa] • 6♂♂, 1♀; same locality and collector as holotype; 26–27 May. 2000; KM-6(♂), 38(♂), 55(♀), 99(♂), 102(♂), TU-749(♂); OPU • 1♂; Mt. Nishime, Kunigami-son; 31 May. 2015; S. Yagi leg.; gen. slide no. KM-133.

#### Diagnosis.

The external morphological character of this new species is quite similar to that of *T. angustella*; the wings of both species are shaded brown apically. However, the two can be differentiated based on the brightness of the color of the apical wing markings. In the new species, the wings feature a huge dark brown area distally, so it can be recognized easily at a glance. Likewise, in the male genitalia, the anellus lobe is a small membranous spherical lobe basally with a delicate, thread-like spine apically. Also, the size of apophyses and shape of the signum in the female genitalia are different to those of *T. angustella*. On the other hand, the male genitalia are quite similar to those of *Thiotricha xanthodora* Meyrick, 1923, which was described from Pyinmana, Myanmar, but differ in terms of the uniformly elongate valva, spherical-shaped and straight phallus. In *T. xanthodora*, the shape of the valva is dilated along 1/3 of its length apically whereas the phallus is slightly rounded basally and twisted forward.

#### Description.

**Male** (Figs 5C, E, 7F). Forewing length 3.3 mm in holotype, 2.5–3.2 mm in paratypes. Wing span 6.2 mm in holotype, 5.6–6.9 mm in paratypes

***Head***: shiny creamy white with appressed scales. Antennae filiform, basal segment rather large and elongate, white, sparsely speckled with brown scales on dorsal surface; flagellum creamy white on dorsal surface before middle, then entirely grayish brown beyond on both surfaces with extraordinarily long and fine cilia ventrally. Labial palps white, long, and recurved; first segment shortest, creamy white with brown scales medially on outer surface; second segment thickened with white scales evenly on both surfaces, as much as 2.5 times the length of the first; third segment as long as second segment, entirely grayish brown, considerably acute and slender.

***Thorax and tegula***: creamy white.

***Legs***: white; forefemur and tibia inwardly suffused with brown and white on outer surface; fore tarsus completely brown; mid femur and tibia entirely white; mid tarsus with brown; hind femur and tibia creamy white, with long, stiff, white bristles until the midpoint anteriorly, brown bristles on upper surface at ca. 1/4 beyond half of its length posteriorly, with white bristles ventrally; all tarsal segments dark grayish in color.

***Forewing***: eleven veins, R_3_ + R_4_ stalked, M_1_ separate, R_5_ absent, anal veins furcate. (Fig. 7F). Forewing rather broad, rounded and slightly pointed apically, ground color creamy white from base along 2/3 of wing, significantly occupied with a huge dark brown or pale-yellow area along costal margin to apex at ca. 1/3 apically; cilia well-fringed and dark brown or yellowish brown before apex to inner base of wing.

***Hindwing***: narrower than forewing, brownish white, pale brown; cilia well-fringed around apex and then white to anterior rim of base.

***Male genitalia***: (Figs 8K, L, 9F) eighth abdominal sternite obtuse, slightly emarginate at the tip, short, and broadened from base toward apex. Uncus swollen and rather small, like a tubercle, with long and short fine setae on its dorsal surface. Gnathos short and stout, slightly flattened posteriorly, then moderately curved apically. Tegumen extremely long and larger than uncus, with a cluster of dense hairs at approximately its midpoint dorsally. Anellus lobe, a small membranous rounded lobe basally, bearing a flexible and weakly sclerotized thread-like spine, reaching toward nearly 2/3 of valva, slightly curved inwardly, a few spines arising around it. Valva simple, uniformly elongate, broad basally, narrowly elongate from base toward apex, with numerous long fine hairs on its ventral surface. Vinculum narrow and elongate, with long and short fine hairs on rim of its surface posteriorly. Saccus somewhat rounded and triangular in shape. Phallus spherical basally, becoming narrow and straight, then slender in distal half.

**Female** (Fig. 5D, F). Forewing length 2.5–3.1 mm. Wing expanse 5.6–6.5 mm. Similar to male.

***Female genitalia***: (Fig. 10F) papillae anales nearly equal in length to posterior apophysis, with long and short fine setae on its entire surface. Apophysis posterioris longer and apophysis anterioris nearly half the length of posterior. Ostium opening close to anterior margin of 8^th^ sternite. Ductus bursae rather broad, uniformly elongate, nearly same length as corpus bursae. Ductus seminalis arising ca. 1/3 of posterior of ductus bursae. Corpus bursae oblong in shape with narrow; signum short and arch-shaped at left side wall of posterior end.

#### Distribution.

Japan (Ryukyus).

#### Etymology.

The name refers to the coloration of the forewing (yellow apically).

#### Host plant.

Unknown.

#### Remarks.

There are two alternative types of wing markings at the distal portion: the brown form collected from Okinawa-jima Island, and the yellow ones from Amami-oshima Island. As mentioned above, individuals with these wing color variations were separately collected from these two islands in the same season. Therefore, this difference may be due to geographical variation.

**Figure 11.**
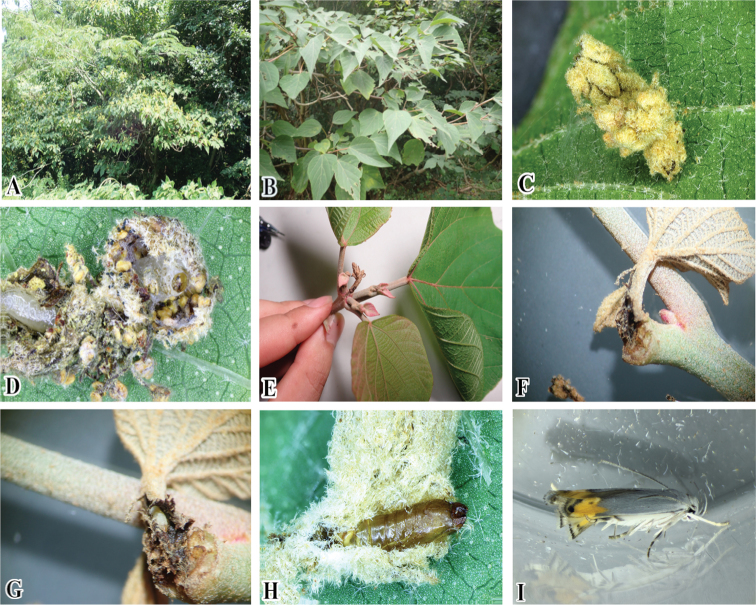
Biology of *Thiotricha chujaensis* (Park, 2016) comb. nov. and its host plant **A** habitat at Kyushu Univ., Fukuoka Pref. **B** host plant, *Mallotus japonica* (Euphorbiaceae) **C** larval portable case made of flower bud attached to the underside of the leaf **D** larva within the portable case **E** a young shoot of host plant **F** Infested part of the shoot **G** Larva inside of the petiole **H** Pupa exuviae **I** Resting posture of adult, lateral view.

## Discussion

### Morphological characteristics of *Thiotricha* and *Cnaphostola*

Meyrick (1885, 1886) stated that the genus *Thiotricha* is characterized by the extraordinarily long ciliation of the male antennae. However, some *Thiotricha* species (e.g., *T. pontifera* Meyrick, 1932 and *T. attenuata* Omelko, 1993) have minute ciliation in the flagellum. Likewise, in the species previously treated as *Cnaphostola*, the rather long ciliation of male antennae is observed in *T. biformis* and *T. venustalis*, while the antennae of *T. angustella* and *T. chujaensis* exhibit extraordinarily long and fine ciliation.

Meyrick (1886, 1918) also defined *Thiotricha* and *Cnaphostola*, respectively, based on the forewing venation. That is, “vein 8 is absent (coincident with 7) in *Thiotricha*, whereas veins 6 and 8 are stalked, and vein 7 is absent in *Cnaphostola*.” However, Meyrick’s description of the forewing venation of *Thiotricha* is incongruent with that of its type species, based on the figure provided by Clarke (1969b) in fig.1a, plate 226. It may be that Meyrick overlooked vein 2 (CuA_2_) and confused veins 6 (M_1_) and 8 (R_4_) of the forewing; they were referred as “veins 7 and 6” in 1886, and “veins 8 and 6” in 1918. Regarding wing venation of the examined specimens in the present study (Fig. 7), vein 2 (CuA_2_) is indistinct and it is likely that it is usually overlooked. In the species previously treated as *Cnaphostola*, M_1_ and R_4_ are stalked in *T. angustella*, *T. chujaensis*, and *T. elaeocarpiella*, but not in *T. biformis*, *T. venustalis*, or *T. flavitermina*. This variation was also pointed out by Meyrick (1886) when describing the characteristics of *Thiotricha*, i.e., vein M_1_ was stalked with R_4_ or separated with R_4_ (explained as “6 rising out of 7 or separate”). Therefore, on the basis of the forewing venation it is impossible to distinguish these two genera.

In terms of male genitalia, *T. chujaensis*, *T. elaeocarpiella*, and *T. flavitermina* have a similar-shaped anellus lobe and gnathos, as some other *Thiotricha* species. Omelko (1984) described three species in *Cnaphostola* based on the similarity of their genitalia, but he did not show that they share diagnostic characters with the type species. Huemer (1993), Tanabe (2013), and Karsholt et al. (2013) mentioned the genital characters of the genus *Thiotricha* (e.g., broad uncus, finger-formed anellus lobe, posteriorly bifurcated sternum VIII, and so on). The type species of the two genera share most characteristics of the head, labial palpus, wing venation, and the finger-like anellus lobe in the male genitalia, although we could not observe the condition of sternum VIII.

Judging from figures given by Clarke (1969a), the type species, *Cnaphostola adamantina* Meyrick, 1918, also has the finger-like anellus lobe in the male genitalia. Moreover, in three species described by Omelko (1984), the male genitalia of both *T. biformis* and *T. angustella* have a finger-like anellus lobe while *T. venustalis* has a slender process. Further, the gnathos shape of these three species is short and stout, as in the type species of *Thiotricha*, *T. thorybodes*, whereas it is long and curved in *C. adamantina* as in some other *Thiotricha* species. That is why, on the basis of the genitalia characters, it is inconclusive whether the type species of *Cnaphostola* belongs to *Thiotricha*, and we therefore refrain from synonymizing the genera here.

In addition, we studied the pupal morphology, and found that characters of *T. chujaensis* and *T. elaeocarpiella* were congruent with those of *T. prunifolivora* as described by Ueda and Fujiwara (2005). However, we point out here that Ueda and Fujiwara (2005) erroneously described that tergal spines are present on the 6^th^ and 7^th^ abdominal segments of the pupa of *T. prunifolivora*. In contrast, these spines are exactly on the caudal and anterior margins of 7^th^ abdominal segment. This condition was also detected in *T. pancratiastis* Meyrick, 1921 and *T. trapezoidella* (Caradja, 1920) in our observations (Kyaw et al. unpubl. data 2019). Although the pupal morphology of *T. angustella* and *T. venustalis* could not be studied, their pupae may also possess such spines which may assist in protrusion of the pupa from their cases; both species were reported to be case bearers (Kogi 2004, 2008).

Based on the reasoning above, we conclude that Japanese species of *Cnaphostola* should be treated in the genus *Thiotricha*, because they share morphological characters of the antenna, labial palps, wing venation and the anellus lobe in the male genitalia with the type species *Thiotricha thorybodes* Meyrick, 1886 and with some other species in that genus (Table 1).

**Table 1.** Morphological characters shared by some Japanese *Thiotricha* species.

Species	Ciliation of male antenna (-) minute (+) rather long (++) extraordinarily long	Labial palps 2^nd^ joint thickened with appressed scales, terminal joint as long as 2^nd^ and acute	Wing venation R4 + M1 (6 and 8) stalked	Sternum VIII bifurcate	Anellus lobe in male genitalia
*T. biformis*	+	+	-	-	+
*T. venustalis* comb. nov.	+	+	-	-	+
*T. pontifera*	-	+	-	-	+
*T. attenuata*	-	+	-	-	+
*T. flavitermina* sp. nov.	++	+	-	-	+
*T. celata*	++	+	-	-	+
*T. pancratiastis*	++	+	-	-	+
*T. synodonta*	++	+	-	-	+
*T. angustella* comb. nov.	++	+	+	-	+
*T. chujaensis* comb. nov.	++	+	+	-	+
*T. elaeocarpiella* sp. nov.	++	+	+	-	+
*T. prunifolivora*	++	+	+	-	+

On the other hand, species of *Thiotricha* are also similar to those of *Polyhymno*. Ueda and Fujiwara (2005) noted a difference in forewing venation: R_5_ is present (5 radial veins are present) in the type species of *Polyhymno*, whereas R_5_ is absent (4 radial veins are present) in the type species of *Thiotricha*. Therefore, it may be appropriate that all species previously treated as *Cnaphostola* should be combined in *Thiotricha*, having 4 radial veins. According to Karsholt et al. (2013), the type species of *Polyhymno* have posteriorly non-bifurcated sternum VIII, a simple valva with reduced anellus lobe, and shorter ciliae on the male antennae. However, we observed that these characters are also shared by some *Thiotricha* species. For example, the sternum VIII is not bifurcate in some *Thiotricha* species, e.g., *T. prunifolivora*, *T. pancratiastis*, *T. pontifera*, *T. indistincta* Omelko, 1993, *T. celata* Omelko, 1993, *T. attenuata*, and *T. synodonta* Meyrick, 1936. Therefore, reexamination of the diagnostic characters of these genera is necessary.

In the present study, although we could not find any definite diagnostic character for separating each genus, we confirmed that the presence of anellus lobe in the male genitalia would provide one of the possible characters for *Thiotricha* which is also shared by all examined species. Furthermore, the pupal morphological characters also support the genus *Thiotricha*. In future studies, a molecular analysis would hopefully clarify the phylogenetic relationships between genera and species and solve the taxonomic problems of the generic delimitation.

### Host utilization and feeding habits of larva

Although Thiotrichinae are known to utilize plants of ten families (all in eurosids), the host range of each species is usually restricted to one genus or a few related genera (Kuroko 1957; Sattler 1982; Huemer 1993; Omelko 1993; Oku 2003; Ueda and Fujiwara 2005; Kaiser et al. 2008; Fujita et al. 2009; Ueda 2011, 2013). Therefore, the host utilization of *Thiotricha elaeocarpiella* is unusual for this subfamily.

**Figure 12.**
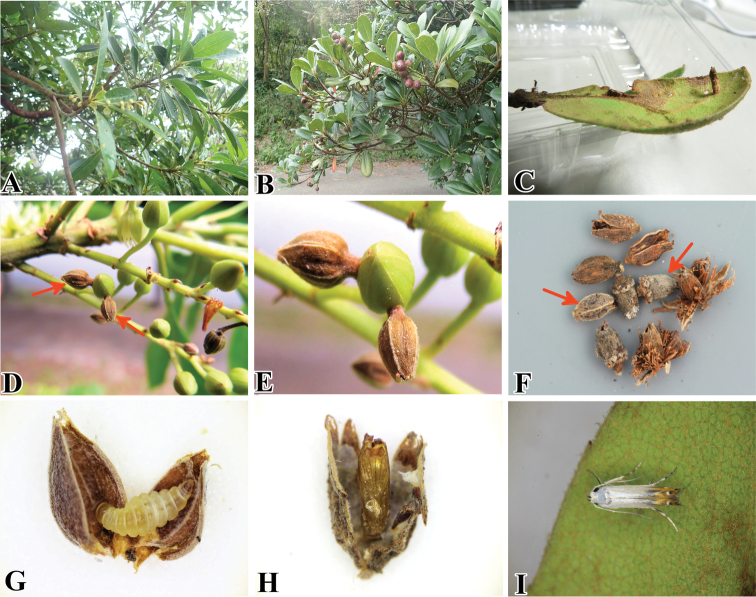
Biology of *Thiotricha elaeocarpiella* sp. nov. and its host plant **A** host plant, *Elaeocarpus zollingeri* (Elaeocarpaceae) **B** host plant, *Rhaphiolepis indica* (Rosaceae) **C** larval portable case made of a young shoot of *R. indica* on the underside of the leaf **D** larval portable cases made with flower buds of *E. zollingeri***E** close up of larval portable cases **F** different types of portable cases. Left arrow indicates the portable case made of *E. zollingeri*, right arrow indicates the portable case made of *R. indica***G** larva within the flower bud **H** pupa exuviae **I** resting posture of adult, dorsal view.

In the present study we observed *T. elaeocarpiella* larvae making portable cases on two different unrelated host plants, *Elaeocarpus zollingeri* (Elaeocarpaceae) and *Rhaphiolepis indica* (Rosaceae), in Okinawa-jima Island and Kyushu. First, we discovered that this species made portable cases with parts of *R. indica* in May 2017 in Fukuoka, Kyushu (Fig. 12C). In the same place in July and August 2017, where *E. zollingeri* grew next to *R. indica*, we also found many differently shaped portable cases made of the flower buds of *E. zollingeri* (*N* = 14) and some portable cases made of *R. indica* under the leaves of *R. indica* (*N* = 2). Secondly, on Okinawa-jima Island, we found many portable cases on *E. zollingeri* in August 2017. Although most of the flower buds were already blooming, we found a certain number (*N* > 20) of portable cases attached to flower buds, most with pupal exuviae inside. We consider *E. zollingeri* to be the main host plant of this species because a great number of portable cases were found on *E. zollingeri* in Kyushu and Okinawa-jima Island.

**Figure 13.**
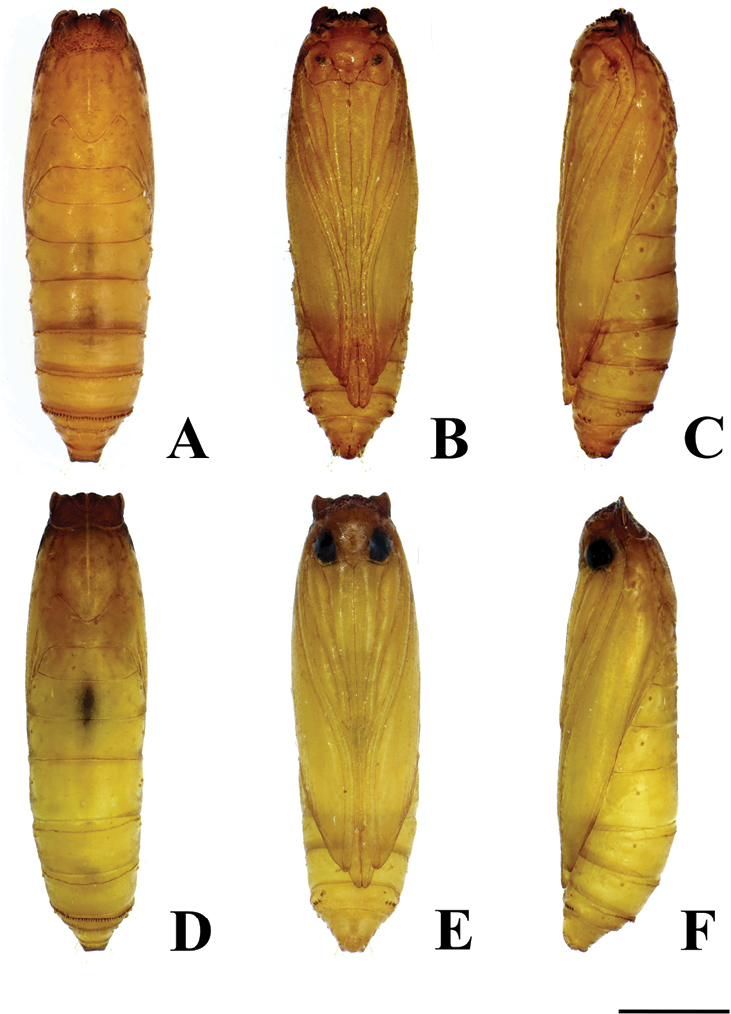
Pupa of *Thiotricha* spp. **A–C***T. chujaensis* (Park, 2016) comb. nov. **D–F***T. elaeocarpiella* sp. nov. **A, D** dorsal view **B, E** ventral view **C, F** lateral view. Scale bar: 0.1 mm.

However, whether the larvae of *T. elaeocarpiella* consumed both plants or accidentally shifted host plants is a controversial matter. Based on shape, the portable case found in May seems to be made of a young shoot of *R. indica*, and we also observed some holes on the leaf near the portable case. These holes are similar to the feeding trace made by *T. prunifolivora*, and the larvae of *T. elaeocarpiella* actually feed on the leaf of *R. indica*. Therefore, we concluded that this species can utilize both of these two plants. In *T. chujaensis*, we found that the larva penetrates the leaf bud (young shoot) of the host plant and then enters entirely and feeds inside (Fig. 11E–G). The last instar larva uses the petiole of the young leaf as a portable case before pupation. Additionally, the larva uses the flower bud at flowering time and makes a portable case on the plant (Fig. 11C, D). After making the portable case, the larva moves and attaches it to the lower surface of a leaf until pupation.

Some *Thiotricha* species showing similar larval feeding habits have been identified. Ueda and Fujiwara (2005) reported that *T. prunifolivora* has three generations per year, and the larvae of each generation have different feeding habits and make a different type of portable case. In the overwintering generation, the hatched larva penetrates the flower bud of the host plant, *Symplocos prunifolia* (Symplocaceae), then uses it as a portable case and attaches the case to the apex of another flower bud with silk. In the first generation, the larva bores into the developing seed and uses it as a portable case, usually accumulating five developing seeds before pupation. In the second generation, the larva bores into the developing seed at first, then after feeding on (usually) two developing seeds, the larva moves to the underside of the host plant and makes several circular holes by feeding. Another species, *T. pancratiastis*, known as a foliage feeder (leaf miner) of *Morella rubra* (Myricaceae), is also reported as a seed predator; the larvae of this species bore into the fruit and, after hollowing it out, utilize it as a portable case. This species probably has two generations annually in Honshu and more in the Ryukyus (Fujita et al. 2009; Ueda 2011, 2013; as *Polyhymno pancratiastis*).

**Figure 14.**
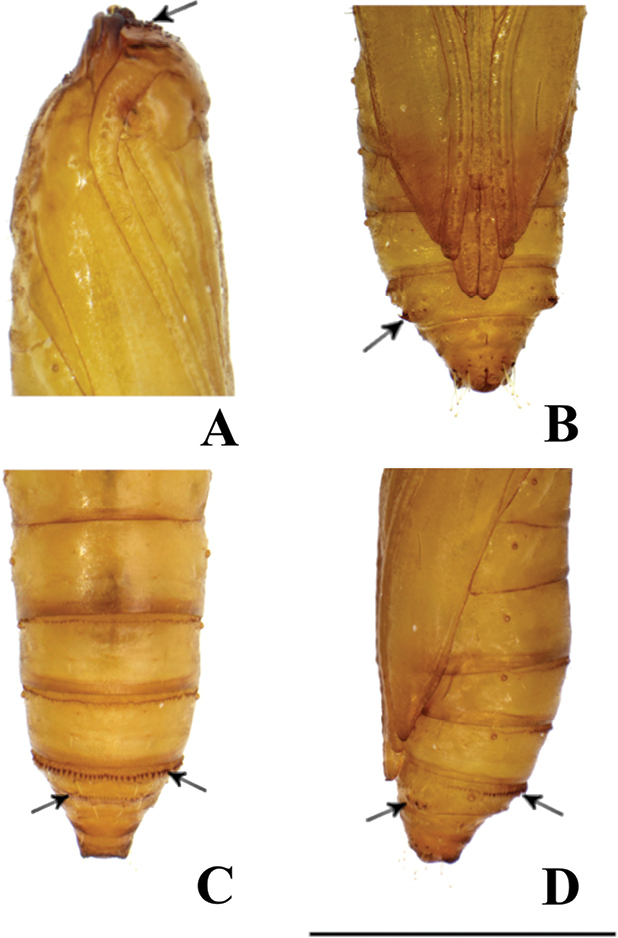
Pupa of *Thiotricha chujaensis* (Park, 2016) comb. nov. **A** head, lateral view Arrow indicates many minute spines on vertex **B** seventh abdominal segment, ventral view Arrow indicates oval pad armed with a row of spines **C** seventh abdominal segment, dorsal view Arrow indicates a row of spines on both anterior and caudal margin **D** seventh abdominal segment, lateral view Arrow indicates oval pad armed with a row of spines and rows of tergal spines. Scale bar: 0.1 mm.

In *T. elaeocarpiella*, *T. chujaensis*, *T. prunifolivora*, and *T. pancratiastis*, the larval feeding mode and behavior are different among generations. These species occur in Honshu, Kyushu, and Ryukyus, have more than two generations a year, and utilize different parts of host plants that grow in temperate climates. On the other hand, *T. venustalis*, *T. angustella*, and some species of *Thiotricha* in the cool climate of the East Palearctic have one generation a year and one mode of feeding (Omelko 1993; Oku 2003; Ueda 2013).

Hence, we presume that the larval feeding mode in *Thiotricha* usually involves the creation of portable cases on host plants, whereas the larvae of *Polyhymno* are leaf-spinners and leaf-webbers (Karsholt et al. 2013). There are some exceptions, such as *T. trapezoidella* and *T. indistincta*; the larvae of *T. trapezoidella* bore into the petiole of Juglandaceae and do not make a portable case (Oku 2003; Ueda 2011, 2013), and *T. indistincta* is a leaf-webber in *Carpinus* spp. (Betulaceae) (Omelko 1993; Oku 2003). In *T. trapezoidella*, however, we confirmed that the larvae make a portable case by cutting the surface of the leaf transversely around the upper tip of the leaf and folding it as a case when the larva is nearly grown to the late instar (Kyaw et al. unpubl. data, 2019). This shows that it is necessary to reinvestigate the larval feeding mode of *Thiotricha* and *Polyhymno* species in detail.

From the results of our taxonomic study together with an exploration of the biology of these species, the evolution of host plant utilization can be elucidated based on species relationships in this genus and its relatives. In future studies, it will be necessary to clarify the biology and DNA sequences of most species.

**Figure 15.**
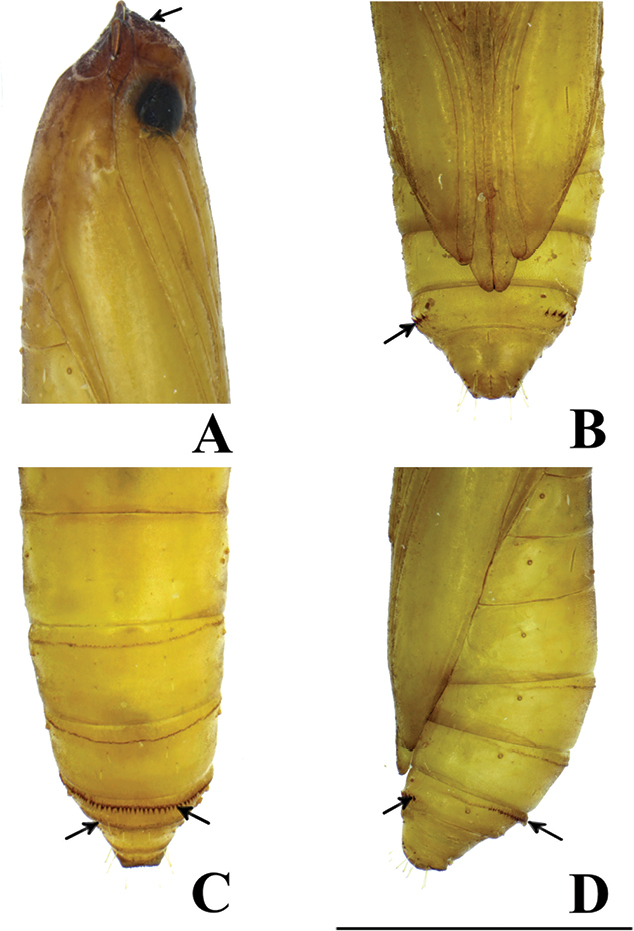
Pupa of *Thiotricha elaeocarpiella* sp. nov. **A** head, lateral view. Arrow indicates many minute spines on vertex **B** seventh abdominal segment, ventral view Arrow indicates oval pad armed with a row of spines **C** seventh abdominal segment, dorsal view Arrow indicates a row of spines on both anterior and caudal margin **D** seventh abdominal segment, lateral view Arrow indicates oval pad armed with a row of spines and rows of tergal spines. Scale bar: 0.1 mm.

## Supplementary Material

XML Treatment for Thiotricha

XML Treatment for Thiotricha biformis

XML Treatment for Thiotricha angustella

XML Treatment for Thiotricha venustalis

XML Treatment for Thiotricha chujaensis

XML Treatment for Thiotricha elaeocarpiella

XML Treatment for Thiotricha flavitermina
